# Clinical Impact of Sphingosine-1-Phosphate in Breast Cancer

**DOI:** 10.1155/2017/2076239

**Published:** 2017-08-22

**Authors:** Junko Tsuchida, Masayuki Nagahashi, Kazuaki Takabe, Toshifumi Wakai

**Affiliations:** ^1^Division of Digestive and General Surgery, Niigata University Graduate School of Medical and Dental Sciences, 1-757 Asahimachi-dori, Chuo-ku, Niigata City, Niigata 951-8510, Japan; ^2^Breast Surgery, Roswell Park Cancer Institute, Elm & Carlton Streets, Buffalo, NY 14263, USA; ^3^Department of Surgery, University at Buffalo Jacobs School of Medicine and Biomedical Sciences, The State University of New York, Buffalo, NY 14203, USA

## Abstract

Breast cancer metastasizes to lymph nodes or other organs, which determine the prognosis of patients. It is difficult to cure the breast cancer patients with distant metastasis due to resistance to drug therapies. Elucidating the underlying mechanisms of breast cancer metastasis and drug resistance is expected to provide new therapeutic targets. Sphingosine-1-phosphate (S1P) is a pleiotropic, bioactive lipid mediator that regulates many cellular functions, including proliferation, migration, survival, angiogenesis/lymphangiogenesis, and immune responses. S1P is formed in cells by sphingosine kinases and released from them, which acts in an autocrine, paracrine, and/or endocrine manner. S1P in extracellular space, such as interstitial fluid, interacts with components in the tumor microenvironment, which may be important for metastasis. Importantly, recent translational research has demonstrated an association between S1P levels in breast cancer patients and clinical outcomes, highlighting the clinical importance of S1P in breast cancer. We suggest that S1P is one of the key molecules to overcome the resistance to the drug therapies, such as hormonal therapy, anti-HER2 therapy, or chemotherapy, all of which are crucial aspects of a breast cancer treatment.

## 1. Introduction

Breast cancer is the most common cancer diagnosis and the second most common cause of cancer death among women in the United States [1]. The majority of deaths due to breast cancer occur after metastasis, and it has become a systemic disease [2]. Breast cancer is known to metastasize to the lymph nodes first, which are a major determinant of prognosis, and forms the basis of the American Joint Commission on Cancer (AJCC) Cancer Staging [2]. A number of signaling proteins, such as vascular endothelial growth factor C and angiopoietins, have been reported to mediate lymphangiogenesis, formation of new lymphatic vessels from the preexisting ones, which plays an important role in lymphatic metastasis [3–5]. In addition to these well-described signaling proteins, we have discovered that a bioactive lipid mediator, sphingosine-1-phosphate (S1P), also plays critical roles in lymphangiogenesis and lymph node metastasis, providing new insights into the mechanisms of cancer progression [6].

Breast cancer is a heterogeneous disease with distinct pathological features and clinical implications [7]. DNA microarray profiling studies on breast cancer have identified distinct intrinsic subtypes: luminal A, luminal B, human epidermal growth factor receptor 2- (HER2-) enriched, and triple-negative [8]. These subtypes are associated with different prognoses and resistance to drug therapy [9–13]. While hormone therapy or anti-HER2 therapies for breast cancer patients with estrogen receptor- (ER-) positive or HER2-enriched subtype are the most successful systemic therapies, only limited therapies are available for patients with the triple-negative subtype. Increasing number of evidence suggested that S1P has different roles in each subtype of breast cancer [14–17].

In this review, we introduce the lipid mediator, S1P, as a new player in breast cancer progression. We will also discuss the potential roles of S1P in breast cancer and the possibilities of S1P signaling as a therapeutic target.

## 2. A Bioactive Lipid Mediator, S1P

S1P is a pleiotropic, bioactive lipid mediator that regulates a number of biological processes, including cell migration, survival, proliferation, angiogenesis, and immune and allergic responses [18–22]. S1P is generated from sphingosine inside the cell [23–27]. Ceramide and sphingosine, the metabolic precursors of S1P, are known to induce apoptosis, thus the balance between them and S1P in the cell has been suggested to function as a rheostat that determines the survival or death of the cell [28].

**Figure 1:**
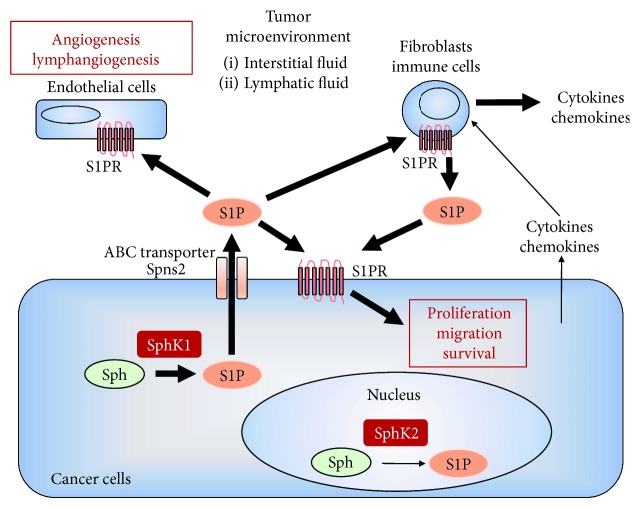
Inside-out signaling of sphingosine-1-phosphate (S1P). S1P is generated from sphingosine (Sph) by sphingosine kinase 1 (SphK1) and sphingosine kinase 2 (SphK2). S1P produced by SphK1 is exported to the extracellular space, such as the tumor microenvironment. S1P secreted to the extracellular space stimulates S1P receptor (S1PR) on cancer cells and brings about cell proliferation, migration, and survival of cancer cells in an autocrine and/or paracrine manner. S1P secreted from cancer cells promotes angiogenesis and lymphangiogenesis. S1P stimulates fibroblasts or immune cells to secrete S1P, which affect cancer cells by inducing cell proliferation, migration, and survival. Cytokines or chemokines secreted from cancer cells also affect fibroblasts or immune cells to secrete S1P. S1P in the interstitial fluid or lymphatic fluid in the tumor microenvironment affects cancer biology.

S1P is generated inside the cells by two sphingosine kinases, SphK1 and SphK2. SphK1 is located in the cytosol close to the cell membrane where its substrate sphingosine resides [29], while SphK2 is localized in specific organelles, such as the nucleus and mitochondria [30] (Figure 1()). S1P produced inside cells by SphK1, but not SphK2, is secreted to the extracellular space by transporters and signals through its receptors on the outside of cells, a process referred to as “Inside-out” signaling [31–33]. S1P produced by SphK2 in the nucleus regulates transcription of various genes expressed in the brain, liver, and kidneys [34–36]. SphK2 in mitochondria produces S1P that interacts with prohibitin 2 to regulate complex IV assembly and respiration [37].

Given its structure, S1P generated inside the cells is unable to freely pass through plasma membrane. S1P export requires transporters such as ATP-binding cassette (ABC) transporters or sphingolipid transporter 2 (Spns2) [14, 38–42]. S1P is exported from mast cells via ABCC1 [43], from astrocytes via ABCA1 [44], from endothelial cells via ABCA1 and ABCC1 [45], and from thyroid carcinoma cells via ABCC1 [46]. Spns2, a member of the major facilitator superfamily of non-ATP-dependent transporters, has been recently discovered to export S1P from cells [40–42]. Importantly, Spns2 was the first S1P transporter discovered to be physiologically functional *in vivo* [42].

After intracellular production by SphK1, S1P is released from the cell and can stimulate any of the five specific G protein-coupled receptors (S1PR1-5), which display tissue-specific expression patterns [14, 38], with each S1P receptor (S1PR) coupled to different G proteins, in an autocrine, paracrine, and/or endocrine manner [47]. The S1PRs are coupled to various G proteins, enabling them to regulate a broad spectrum of downstream signaling pathways and numerous biological processes [48–51].

S1P levels are tightly regulated by the balance between its synthesis and degradation. S1P can be dephosphorylated back to sphingosine by two specific S1P phosphatases (SPP1 and SPP2), which belong to the family of magnesium-dependent, N-ethylmaleimide-insensitive type 2 lipid phosphate phosphohydrolases that reside in the endoplasmic reticulum [32, 52, 53]. S1P can also be irreversibly degraded by a pyridoxal phosphate-dependent S1P lyase to hexadecenal and phosphoethanolamine, with the latter subsequently being reused for the biosynthesis of phosphatidylethanolamine [31].

## 3. S1P and Breast Cancer Progression

**Figure 2:**
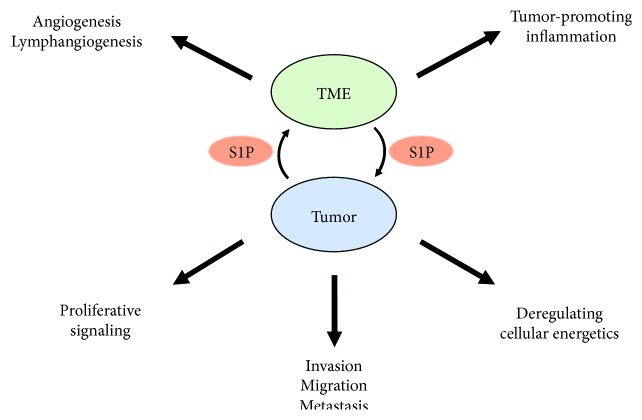
The roles of sphingosine-1-phosphate (S1P) in the interaction between tumor and tumor microenvironment (TME). S1P produced by tumor cells and the TME promote various pathological processes related to cancer progression.

The tumor microenvironment (TME) is a determining factor for cancer biology and progression. The TME comprises not only cancer cells, but also the surrounding blood vessels, immune cells, other stromal cells with signaling molecules, and the extracellular matrix and interstitial fluid, which are now known to play important roles in tumor progression [54–56]. Determining the interaction between cancer and noncancer cells in the TME is essential to understanding the mechanisms underlying cancer progression and metastasis. Cancer cells provide bioactive molecules, such as cytokines, chemokines, and lipid mediators to the TME that influence cancer progression. Noncancer cells in the TME, such as blood vessels, lymphatic vessels, and inflammatory cells, also release bioactive molecules that influence cancer progression. S1P is now emerging as a key regulatory molecule in breast cancer through its ability to promote cell proliferation, migration, angiogenesis, and lymphangiogenesis (Figure 1). Further, S1P secreted to the extracellular spaces, including the interstitial fluid and lymphatic fluid, has been suggested to be important for metastasis by stimulating S1P signaling [30, 57]. In this section, we describe the roles of S1P in the TME and tumor progression (Figure 2).

Angiogenesis, the development of new blood vessels from preexisting vessels, determines the rate of growth and progression of cancer by providing oxygen, nutrition, and conduits for cancer cells for invading cells to metastasize [58, 59]. The neutralization of extracellular S1P with an anti-S1P antibody *in vitro* and in an animal model showed significant inhibition of angiogenesis, tumor growth, and metastasis, further confirming the dominant role of extracellular S1P in angiogenesis [60]. Tumors with upregulated SphK1 may themselves be a key source of S1P according to the data from mouse models and human patient samples [6, 61–64]. However, endothelial cells can also synthesize and release endogenous S1P [45].

In contrast to angiogenesis, few studies so far have examined the involvement of S1P signaling in lymphangiogenesis [65]. S1P can induce lymphatic endothelial cell (LEC) tube formation in an S1PR1-dependent manner [4, 66]. Moreover, angiopoietin-2-induced lymphangiogenesis *in vitro* was suppressed by an SphK1-specific pharmacological inhibitor, suggesting cross talk between angiopoietin-2 and S1P signaling pathways [6]. LEC-specific deletion of SphK1 in the SphK2 knockout mouse inhibited lymphatic vessel maturation, indicating that SphKs and S1P in LECs are required for proper development of lymphatic vessels [3].

Spns2 is an S1P transporter that plays a role in the regulation of the lymph node and lymphatic fluid S1P levels and consequently influences lymphocyte trafficking and lymphatic vessel network organization [67]. Spns2-deficient mice showed an aberrant lymphatic vessel network that appeared collapsed, with reduced numbers of lymphocytes. Levels of S1P were increased in the lymphatic fluid from Spns2-deficient mice as well as in specific tissues, including lymph nodes, and interstitial fluid.

Utilizing our newly established murine syngeneic orthotopic metastatic breast cancer model and mass spectrometry, we found that levels of S1P gradually increased both in tumors and in the circulation and correlated with tumor burden [6]. Further, treatment of tumor-bearing animals with the specific SphK1 inhibitor, SK1-I, reduced S1P levels in the tumor and in circulation and greatly reduced the tumor burden of the primary tumor, lymph node, and lung metastases. Importantly, inhibiting SphK1 significantly decreased S1P levels in serum and tumors in these mice, and both angiogenesis and lymphangiogenesis were suppressed, not only around the primary tumor but also in lymph nodes that were distant from the tumor [6]. Furthermore, lymph node and lung metastases were significantly suppressed. These results indicate that S1P plays a key role, not only in tumoral lymphangiogenesis, but also in lymph node lymphangiogenesis, which may actively promote metastasis via the lymphatics [6].

An S1P gradient with high S1P concentrations in the blood and lymphatic fluid circulation plays an important role in immune cell trafficking [67–71]. We established a convenient method for the collection and measurement of sphingolipids in the lymphatic fluid. The lymphatic fluid was absorbed onto filter paper after incision of cisterna chyli in murine models, and S1P levels were determined by mass spectrometry [72]. Levels of S1P in the lymphatic fluid are lower than those in blood and higher than those in lymph nodes in agreement with a previous study [72]. Levels of S1P in the lymphatic fluid as well as lymph node were significantly increased in SphK2 knockout mice compared to littermate controls. These results suggest that SphK2 and/or SphK1 regulate the levels of S1P in the lymphatic fluid.

The interstitial fluid that bathes the tumor and stromal cells is an important part of the TME, not only as the initial route of metastasis, but also as a supplier of factors that promote tumor metastasis. The role of S1P in the TME, particularly in the interstitial fluid, has not been well studied due to a lack of efficient methods for collecting and quantifying levels in the interstitial fluid. We developed simple and reproducible methods to measure the levels of sphingolipids including S1P in small volumes of the interstitial fluid from healthy mammary glands and tumor using a modified centrifugation method combined with mass spectrometry [57]. In mice with a deletion of SphK1, but not SphK2, levels of S1P in the interstitial fluid from the mammary glands were greatly reduced. Levels of S1P in the interstitial fluid from the mammary tumors were reduced when tumor growth was suppressed by oral administration of the potent S1P receptor functional antagonist, FTY720 (fingolimod; 2-amino-2-[2-(4-octylphenyl)ethyl]1,3-propanediol) [57]. Thus, S1P secreted from tumor cells to the interstitial fluid may play an important role of metastasis by stimulating S1P signaling in the TME [57]. Further, in breast cancer patients, we examined the levels of sphingolipids in the interstitial fluid from breast tumor tissue and normal breast tissue from two different areas in each patient and determined levels of sphingolipids in the fluid. We revealed that S1P levels were significantly higher in the human breast tumor tissue interstitial fluid than in the normal breast tissue interstitial fluid [57]. Thus, the observations made in animal models are applicable to human patients.

## 4. Clinical Impact of S1P in Human Breast Cancer Patients

The interaction between cancer cells and the TME is now considered key to understanding the mechanisms of how cancer progresses and metastasizes. Although numerous *in vitro* and *in vivo* studies have reported the importance of S1P in breast cancer progression, the evidence in human breast cancer patients had been limited until recently. We have determined the levels of S1P in breast cancer and normal breast tissues from surgical specimens and compared the difference in levels of S1P between those tissues [73]. S1P levels in the human breast cancer tissue are significantly higher than those in the normal breast tissue. Of note, there was a correlation in the levels of S1P between the breast cancer and normal breast tissues, which implies that S1P produced by tumor cells affect surrounding normal breast tissue. Taken together, S1P can be considered an important player in the interaction between cancer and the TME.

We also investigated ceramide metabolism in breast cancer patients and showed that ceramide levels in the human breast cancer tissue are significantly higher than those in the normal breast tissue [73]. Interestingly, however, there was no correlation in the ceramide levels between the breast cancer and normal breast tissues [73]. It has been reported that ceramide has a distinct role in cell survival and death. This finding indicates that ceramide production in tumor tissue occurs independently from the surrounding normal breast tissue. Further investigation is needed to determine the roles of ceramide in human breast cancer biology.

We have also quantified the levels of S1P in human blood and tissue samples from breast cancer patients utilizing mass spectrometry. Importantly, serum S1P levels were significantly elevated in stage IIIA breast cancer patients who had developed lymph node metastases, compared with age- and ethnicity-matched healthy volunteers [6]. More recently, we found that patients with lymph node metastasis showed significantly higher levels of S1P in breast cancer tissue than patients with negative nodes [74]. These findings revealed that S1P produced by breast cancer tissue affects lymph node metastasis in human patients, consistent with the observations in animal models. Research to clarify the role of S1P in cancer progression has now evolved from an experimental phase to a translational phase. More data from the clinical setting is now needed to translate the previous theories of how S1P promotes cancer progression based on *in vitro* and *in vivo* models to human breast cancer care.

## 5. S1P as a Therapeutic Target for Breast Cancer Patients

Breast cancer is treated based on the intrinsic subtype as described above. Pharmacological inhibition of 17*β*-estradiol (E2) production or E2 binding to the ER is an effective treatment for ER-positive patients. Although HER2-positive breast cancer is known to be aggressive, and patients with HER2-positive breast cancer had a poorer prognosis, anti-HER2 therapy, such as trastuzumab, pertuzumab, and TDM-1, which are recombinant antibodies that target HER2-positive cancer cells, have dramatically improved survival of patients with this subtype.

In contrast to ER-positive or HER2-positive breast cancers, ER-negative and HER2-negative breast cancers do not have specific therapies, which are one of the reasons that the latter type often demonstrates earlier disease recurrence and poorer prognosis. Any cancer subtype has a potential to gain resistance to a particular therapy. It has been reported that there is cross talk between the S1P signaling pathway and the E2 or HER2 signaling pathways. Thus, S1P may have a role in acquisition of drug resistance [75].

**Figure 3:**
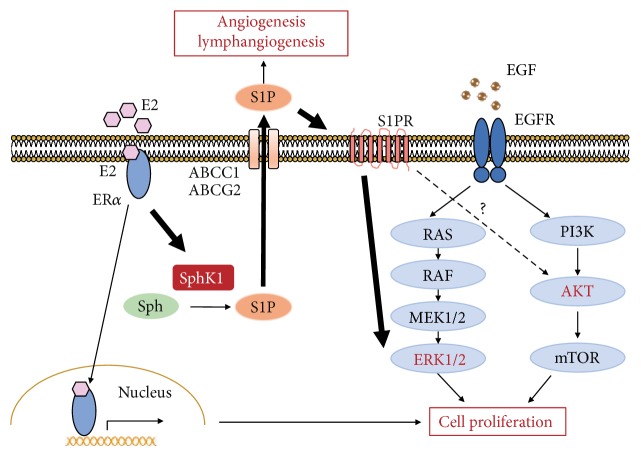
The role of sphingosine-1-phosphate (S1P) in estrogen receptor- (ER-) positive breast cancer. Binding of estradiol (E2) to ER-*α* stimulates sphingosine kinase 1 (SphK1) to produce S1P from sphingosine (Sph). S1P is exported out of cancer cells via ATP-binding cassette (ABC) transporter, such as ABCC1 and ABCG2. This S1P activates S1P receptor (S1PR) of cancer cells to stimulate ERK1/2, which contributes to cell proliferation. S1P may also affect the PI3K pathway by activating AKT.

ER status is an important prognostic factor with ER-positive breast cancer patients having a better prognosis than ER-negative breast cancer patients. We reported the contribution of the S1P pathway to E2 signaling. Binding of E2 to ER stimulates release of S1P via ABC transporters, ABCC1 and ABCG2 [14]. This S1P in turn binds to and activates S1P receptors to stimulate ERK1/2 leading to downstream signaling events important for breast cancer proliferation, progression, and invasion. We showed that E2 induces export of S1P via ABCC1 and ABCG2 transporters, which may contribute to the nongenomic signaling of E2 important for breast cancer pathophysiology [14]. Higher levels of SphK1 were found in ER-negative breast cancer, which are known for their higher proliferative activity [63]. It has also been shown that SphK1 expression correlates with poor prognosis of breast cancer patients [63]. Even in ER-positive breast cancer, higher expression of SphK1 correlated with poor patient survival rates and was associated with the development of tamoxifen resistance and earlier disease recurrence while on tamoxifen [76, 77]. Therefore, SphK1 expression levels in combination with ER status are proposed to be good biomarkers to predict response to tamoxifen [77] (Figure 3).

HER2 overexpression is a major determinant of breast cancer progression. S1PR4 stimulates the ERK1/2 pathway via a HER2-dependent mechanism in ER-negative MDA-MB-453 breast cancer cells [15]. High expression of S1PR4 and SphK1 is associated with shorter disease-specific survival in ER-negative breast cancer patients, highlighting the important role for S1PR4 and SphK1 in ER-negative breast cancer progression [78]. We found that breast cancer tissue S1P levels were lower in those with HER2 overexpression/amplification [74]. Considering that both HER2 and SphK1 are strong activators of survival signaling pathways such as MAPK, and HER2 signal is a strong autonomous signal, it is tempting to speculate that negative feedback suppresses activation of SphK1 in HER2-positive breast cancer. Further study is needed to determine the relationship between S1P signal and HER2 overexpression/amplification.

Patients with triple-negative breast cancer have a poor prognosis relative to other breast cancer subtypes. LM2-4 cells that gained lung metastatic phenotype from primary triple-negative MDA-MB-231 breast cancer cells showed a requirement on SphKs/S1P signaling for cell growth, survival, and cell motility. PF-543, a selective, potent inhibitor of SphK1, attenuated epidermal growth factor-mediated cell growth and survival signaling through inhibition of AKT, ERK, and p38 MAP kinase pathways in LM2-4 cells, but not in the parental MDA-MB-231 human breast cancer cells [16]. These observations highlight the importance of SphKs/S1P signaling in metastatic triple-negative breast cancers and targeted therapies [16].

The roles of ATP-binding cassette transporters, such as ABCC1, ABCC11, and ABCG2, in breast cancer patients have been reported. For instance, ABCC1 and ABCG2 have been associated with chemoresistance in breast cancer [79]. Further, ABCC1 and ABCG2 are highly expressed in core basal subtype, which is one of the most aggressive breast cancer subtypes, while ABCC11 is highly expressed in the HER2 or core basal subtypes [80]. Moreover, ABCC1 or ABCC11 expression is associated with shorter disease-free survival [80]. Taken together, ABC transporters are highly expressed in aggressive breast cancer subtypes, and tumor ABC transporter expression is associated with poor prognosis.

Several agents targeting S1P signaling have been tested in preclinical models [81, 82]. FTY720 is a novel immunosuppressive agent that shows structural similarity to sphingosine and is intracellularly phosphorylated by SphK2, but not SphK1 [83, 84]. FTY720-P, the phosphorylated form of FTY720, binds to S1PR1 in lymphocytes and/or endothelial cells, which causes the inhibition of lymphocyte trafficking into the circulating blood and the accumulation of lymphocytes in secondary lymphoid tissues [85–87]. FTY720-P is transported through the same pathway as S1P [88]. Further, FTY720 has been approved by the US Food and Drug Administration (FDA) for multiple sclerosis [31]. SK1-I is a selective SphK1 inhibitor [89, 90]. We found that BML-258, one of the SK1 inhibitors, reduced S1P levels in the tumor and in circulation, and greatly reduced the size of the primary tumor, lymph node, and lung metastasis in animal model [6]. The anti-S1P mAb, sonepcizumab, and its murine counterpart, sphingomab, substantially reduced tumor progression, and, in some cases, eliminated measurable tumors [91, 92]. Antibody-mediated neutralization of extracellular S1P could result in a reduction of tumor volumes and metastatic potential, as a result of inhibition of new blood vessel formation [60, 93]. Further studies are needed if new therapies are to be developed for cancer patients based on S1P pathways.

## 6. Conclusion

In this review, we discussed the roles of S1P in breast cancer progression. S1P secreted from cells interacts with the TME, which has been suggested to be important for metastasis. Importantly, recent translational research has demonstrated an association between S1P levels in human breast cancer patients and clinical outcomes, which highlights the clinical importance of S1P in breast cancer. We suggest that the S1P pathway could be an important therapeutic avenue to overcome the resistance to drug therapies, such as hormonal therapy, anti-HER2 therapy, or chemotherapy, all of which are crucial aspects of breast cancer treatment.

## Abbreviations

ABC: ATP-binding cassette
E2: 17*β*-estradiol
ER: Estrogen receptor
HER2: Human epidermal growth factor receptor 2
LEC: Lymphatic endothelial cell
S1P: Sphingosine-1-phosphate
SphK: Sphingosine kinase
Spns2: Sphingolipid transporter 2
SPP: S1P phosphatase
TME: Tumor microenvironment.
